# Book Reviews

**Published:** 1804-04-01

**Authors:** 


### CRITICAL ANALYSIS

OF THE

### RECENT PUBLICATIONS

ON THE

### DIFFERENT BRANCHES OF PHYSIC, SURGERY, AND MEDICAL PHILOSOPHY.

*The Anatomy and Surgical Treatment of Congenital and Inguinal Hernia; by* Astley Cooper, F.R.S. &c. &c.

( Continued from pp. 279—282. )

In the ninth chapter the author treats of the circumstances to be considered previous to the operation. Every practitioner has had reason to lament that so much precious time is often wasted in fruitless attempts at reduction by the taxis long after every reasonable able prospect of success has vanished; and a few instances to the contrary do not invalidate the general rule of operating as soon as possible after this period, when it may still be done with probable safety and success. The death of a late highly esteemed nobleman is understood to be one of the many melancholy instances in proof of this rule of practice, which the author enforces with due attention.

Mr. C. observes that there is no very obvious criterion to determine the latest time to which the operation may be safely delayed. Hiccough, though a common symptom of mortification, is not always present in this state, and often occurs before it is established, as has been proved by many successful operations at the Hospitals in the Borough after hiccough has appeared. Soreness of the belly on pressure, succeeding great tension of the abdomen, Mr. C. thinks the least fallible sign of inflammation having proceeded to a fatal extent.

The operation itself is the subject of the next and very important chapter, part of which we shall give to our readers on account of the valuable, and in some degree original, observations which it contains. After describing the method of making the incisions through the integuments, and of opening the sac so as to expose its contents, the main object of the operation, namely, to relieve the stricture, is the next care of the surgeon. By carrying his finger up the hernial sac, he will find the stricture in one or other of the three following situations.

"First, at the abdominal ring.

"Secondly, above the ring, from one inch and a half to two inches, and inclining outwards toward the spinous process of the ilium.

"Thirdly, in the mouth of the hernial sac.

"If the stricture is owing to the pressure of the columns of the tendon which form the abdominal ring, it is then to be divided in the following manner. The surgeon passes his finger into the sac, as far as the stricture, and then conveys a probe-pointed bistory on the fore part of the sac, and, insinuating it within the ring, cuts through it in a direction upwards opposite to the middle of the sac, and to an extent proportioned to the size of the tumour. . . .

"It is best to divide the stricture by passing the knife between the ring and the sac, as a larger portion of peritoneum is thus left uncut, and the cavity of the abdomen is afterwards more easily closed.

"The direction given to the knife in dilating the stricture has been usually upwards and outwards towards the spine of the ilium, but I prefer doing it directly upwards for the two following reasons: First, as the higher aperture must only be dilated directly upwards, it is better that the surgeon should have one general rule for the use of the bistory, applicable to every case of inguinal hernia, than to be perplexed in the operation by a variety of directions; and, secondly, the division of the tendon in this direction weakens the the abdomen less than upwards and outwards, because, as the cord passes towards the abdomen in that direction, and the hernial sac is parallel to the cord, a dilatation in that course takes off the resistance which the tendon would otherwise make to any future descent. When the ring is divided directly upwards, the upper column of tendon which forms it, is cut; when it is dilated upwards and outwards, the transverse fibres uniting the columns are divided."

The second situation of stricture is above the ring, towards the spinous process of the ilium, at the orifice whence the hernia first quits the abdomen; and the constriction is occasioned by the tendon of the transversalis passing over the hernia, and the resistance of the edge of fascia under it. Here the Author lays down the following direction.

"The surgeon passes his finger up the sac and through the abdominal ring, till he meets with the stricture; he then introduces the probe-pointed bistory with its flat side towards the finger, but anterior to the sac, and between it and the abdominal ring, his finger being still a director to the knife. Thus he carries the knife along the fore part of the sac until he insinuates it under the stricture formed by the lower edge of the transversalis and internal oblique muscles, and then turning the edge of the knife forwards, by a gentle motion of its handle he divides the stricture sufficiently to allow the finger to slip into the abdomen; the knife is then withdrawn, with its flat side towards the finger, as it was introduced, to prevent any unnecessary injury to the parts."

The direction of this [dilation being straight] upwards, scarcely any risk is incurred of cutting the epigastric artery.

The third seat of the strangulating stricture is a thickening of the mouth of the sac itself, but which the author considers as a rare occurrence.

The chapter concludes with remarks on the treatment of the omentum, in which the Author condemns the custom of applying a ligature round it, to make it slough away, as has sometimes been practised.

The succeeding chapter, on mortification of the intestine, contains some discriminating practical remarks and original experiments, in confirmation of them, which deserve notice. The treatment is the following. "As the intestine, when mortified, cannot be returned into the cavity of the abdomen, the surgeon is to consider in what manner he is to proceed, to save his patient from that most miserable state of existence which is produced by an artificial anus. In forming his judgment upon this subject, he will be directed by the state in which the part is found.

"If a small hole only has been produced, the intestine should be returned into the abdomen, excepting that portion of cylinder in which the hole exists. A needle and ligature should be passed through the mesentery at right angles with the intestine, to prevent it including the branches of the mesenteric artery which supply that part of the intestine, and then through the mouth of the hernial nial sac; and tying the thread, the intestine becomes confined to the mouth of the hernial sac, and the fæces pass readily from the opening by the wound, but will in part take their course by the rectum. As granulations arise and the wound becomes closed, the opening in the intestine is gradually shut, and an artificial anus is effectually prevented."

This first variety is illustrated by a successful case.

But when the whole cylinder of intestine is mortified, Mr. C. advises that "the mortified part of the intestine should be cut away, and the ends brought in contact, and confined by means of four ligatures."

Some experiments follow. The author divided entirely the intestine of a dog, introduced within it a cylinder of isinglass, and made three sutures upon it, so as to unite the divided bowel. In three days the animal had stools; and on the sixteenth day, when he was killed, the union was found complete. The same was repeated without the isinglass, and with equal success. In both these experiments, when the intestine was returned, the ends of the ligatures were left hanging out of the wound.

Other experiments are introduced, which were made by Mr. Thompson, Lecturer in Surgery in Edinburgh. In the first of these the intestine of a dog was divided, then sewn up as before, but the ligatures returned entirely into the abdomen, and the external wound closed. On the tenth day the animal was killed in health; the intestine was perfectly united, the traces of the ligatures partly obliterated on the outside, and some of the threads had disappeared; and as they had passed through into the intestine, they had doubtless been discharged by stool. A second experiment, in which more time was taken, gave a similar result.

These experiments are valuable, as they shew the comparative safety of returning a ligature of the intestine into the cavity of the belly, though the author prefers retaining the ends of the threads at the external wound. The subjects of the experiments, however, were animals in previous health; and the author himself admits, that a suture of the intestine in hernial cases sometimes brings on such dangerous symptoms, that an immediate removal of the threads is requisite This interesting chapter concludes with further experiments and remarks on the union of wounds in the intestine.

The treatment after operation is that usually adopted. It has been proposed by some to effect a radical cure during the operation, by removing the sac either with the knife or by ligature. Mr. C. shews the danger and usual impracticability of this proceeding; the danger chiefly attends the ligature from the well known tendency of inflammation to extend through membranes in all their extent from a single morbid point.

A separate chapter is made on the treatment of *very large Herniæ*, chiefly as leading to an improvement in the mode of operation. This is before mentioned in describing the common operation, tion, and is repeated here as particularly applicable. It is to return the herniæ merely by dilating the stricture, *without* opening the sac; a practice which the author appears first to derive from the eminent Professor at Anatomy at Edinburgh, "Dr. Monro, of Edinburgh, to whom it would be ingratitude not to acknowledge the obligation I feel for the instructions conveyed in his Lectures, has strongly urged the propriety of dilating the abdominal ring, without opening the sac, in hernia in general; and I feel convinced that this operation will be gradually introduced into general practice, when it has been fairly tried, and found, if performed early, to be free from danger, and attended with no unusual difficulty."

The propriety of this practice the author states to be peculiarly applicable to very large herniæ, on account of the accumulated difficulty of returning the hernial contents, from their frequent adhesion, and from the habitually diminished size of the abdomen, which expose the intestine, if the sac is opened, to a degree of unavoidable injury from the operator that often proves fatal. Two useful cases are given in illustration of each method; the one successful, the other fatal.

Very small hernia are next considered. These are in fact *incipient* hernia, or protrusion of the intestine through the upper abdominal opening, extending only to the ring, and not descending into the scrotum.

The tumour produced by this variety is very small, not more than by an enlarged gland, and hence they are sometimes overlooked, and the patient dies as is supposed from some bowel complaint unconnected with surgery. The operation for this hernia only requires the incision to be made above the ring, towards the spinous process of the ilium.

Sometimes the sac itself has been returned, when being recent, it has contracted no considerable adhesion to the adjacent tendons.

An important variety of hernia is next described. It is a descent of the intestine *directly opposite the abdominal ring*, and not obliquely from the upper abdominal opening in the course of the spermatic cord. This variety is really what common inguinal hernia was formerly supposed to be. It is thus described "The abdominal ring is closed towards the abdomen by the tendons of the internal oblique and transversalis muscles. The lower parts of these tendons are inserted into the pubis, and connected with the fascia which passes upwards from the external oblique muscle at Poupart's ligament. If this tendon is unnaturally weak, or if from malformation it does not exist at all, or from violence has been broken, a protrusion of the viscera may then take place immediately behind the ring. . . . . . . . . . . . Below the abdominal ring the appearance of this tumour differs from that of common bubonocele in being situated nearer the penis, and the spermatic cord passes on its outer side, instead of its posterior part, particularly at and above the abdominal ring. Above the abdominal ring the sac passes directly upwards, so that no part of it takes the usual oblique direction direction towards the anterior superior process of the ilium, but rather the contrary direction towards the navel. Examined by accurate dissection its course is as follows: The sac first protrudes between the fibres of the tendons of the transversalis, nearly an inch directly above the ring. It then passes under the lower edge of the tendon of the internal oblique muscle. The epigastric artery runs upon the outer side of the hernial sac. The spermatic cord has no connection with it above the ring. The hernia then emerges from the abdominal ring, the spermatic cord being on its outer side, and it is covered with the facia given off by the tendon of the external oblique but not by the cremaster muscle."

The most important circumstance in this variety of inguinal hernia is the situation of the epigastric artery, which is precisely the reverse of that in ordinary bubonocele; in the common disease the epigastric artery runs on the inner side of the tumour, or nearer to the linea alba; in this variety it is situated on the outer side: Consequently, the dilatation of the stricture upwards and *outwards* will in this instance divide the artery, an accident generally fatal from the hæmorrhage into the cavity of the abdomen. To avoid it, Mr. C. observes that it would be the best in these cases to dilate *inwards*, if the nature of the variety is fully ascertained; but as the diagnosis is sometimes difficult, and as too much must not be left to the discrimination of every surgeon who may be called to operate on hernia, and because cutting inwards would incur the same risk of dividing the artery if the bubonocele were of the more common species, he advises to dilate here, as in every other instance, *directly upwards*, which will avoid this danger in either case.

Very frequent dissection has convinced Mr. C. that the above-mentioned variety of hernia, emerging from the abdomen opposite the ring, is by no means uncommon.

Nothing particular occurs in the Chapter on Hernia in the Female.

The volume concludes with a very instructive chapter on Congenital Hernia, in which a curious variety is given by Mr. Forster, surgeon to Guy's Hospital, of a hernial sac existing within the tunica vaginalis.

The plates contained in this volume form so large and important a part of its contents, that they require to be individually noticed. This we shall do in a few words, premising, that to the elegance of beautiful execution on the part of Mr. Heath, the engraver, they unite the advantage (in a scientific work infinitely more important) of being drawn apparently with perfect fidelity by Mr. Kirtland, of the natural size, from preparations, as we are informed in the Preface, actually in the author's possession, or in the Museum at St. Thomas's Hospital.

The first plate is purely anatomical, shewing the healthy structure of the parts immediately connected with hernia, particularly the insertions of the oblique muscles, Poupart's ligament, the valvular structure of the upper abdominal opening, and the passage of the spermatic cord through it down to the ring. The

The second plate is an internal view of the same opening, and the relative situations of the spermatic, epigastric and iliac arteries.

The representation of hernia succeeds in the next; on one side large and old, filling the scrotum; on the other incipient, or of the kind described in the chapter on small hernia, appearing like an enlarged gland.

The next, an internal view of the same, shewing the effect of gradual enlargement in old bulky herniæ, to bring the upper abdominal opening nearly opposite to the ring.

In the next, several smaller figures are given, shewing the relative situation of the epigastric and spermatic vessels, the gradual progress of hernia, septa dividing the hernial sac, the effect of the improper use of trusses in only partially obliterating the disease, and some singular varieties.

In the following plate are described the form and application of trusses, an incipient hernia just quitting the abdomen, and a coloured figure (the only one of this kind in the volume) representing the peculiar livid hue of mortified intestine.

The next plate, which is admirably instructive, exhibits in the same subject, a hernia on each side; on the left, situated at the outer side of the epigastric artery, and on the right, at the inner side. The danger of wounding this artery and the caution requisite to avoid this accident are clearly seen.

An internal view of the same preparation follows.

A still more complicated case of hernia in the same subject is represented in the following plate.

The next is remarkable for the number of herniæ in the same subject, with a complication which could not have been discovered but by dissection.

The eleventh and last plate, containing several figures, exhibits in one that singular variety of inguinal hernia found by Mr. Forster; in the others, illustrates different points in the operation, particularly that of returning the hernia without opening the sac, so strongly recommended in the foregoing part of the work.

The style is a simple enunciation of facts, clear and unaffected. Almost every thing relating to the history of opinions and discoveries in this disease is omitted; the author has appeared desirous to incur a personal responsibility for the accuracy of every fact and assertion, and to confine himself to the results of a multitude of dissections, of which actual demonstration exists in one or two cabinets of anatomy, or to the records of numerous operations of which living witnesses remain. Under a plan thus circumscribed, to have made so valuable an accession to the kindred arts of anatomy and surgery, will be a lasting testimony to the knowledge, professional skill, and unsparing industry of the Author, worthy of the noble field of observation in which they are habitually employed.

This volume is dedicated with peculiar propriety to the eminent Teacher whose lectures on anatomy and surgical skill have for so many years enriched the School of St. Thomas's Hospital.

---

*A Synoptical*

*A Synoptical Table of Diseases, exhibiting their Arrangement in Classes, Orders, Genera and Species, designed for the Use of Students;* By A. Crichton, M. D. F. R. S, &c. (This is published in one very large sheet.)

The Author has prefixed a short Introduction, in which he assigns the reasons which led him to this publication. Many of them have doubtless struck other Teachers, who have felt the impossibility of adhering strictly to any system of Nosology before published.

"The great object to be obtained by an artificial arrangement of the matter of any science, Dr. C. observes, is practical utility. In regard to Medicine, this utility is of two distinct kinds; *first,* the facilitating the business of instruction, and *secondly,* the assisting the practitioner with such hints and views, as well regulated analogy always yields.

"In all sciences, analogy or similitude is the great principle on which arrangement takes place. This analogy or similitude may have reference to different things, not only in different sciences, but even in the same science. In regard to diseases, it may refer either to the phenomena, or to the nature or cause of diseases, or to their seat, or cure; or it may have a mixed reference to all of these. It may easily be proved, however, that if an arrangement of diseases is attempted to be exclusively founded on any one of these grounds, it must be defective. Similarity of symptoms, it is well known, often occurs in diseases of an opposite nature, and an arrangement exclusively founded on such a principle, would connect diseases which had no other natural analogy than external character, and would rather tend to injure than promote the views of practice.

"The seat of diseases has been made the basis of nosological arrangement in a few instances, but it is evident that such a method can only be useful as an assistant to memory, otherwise diseases will frequently be brought together which have no real analogy with each other, either with respect to their causes or cure. No system of arrangement has hitherto been founded on the method of treatment alone, and indeed, considering the state of Medicine, it is evident that it must not only be an imperfect, but in many instances, an hypothetical method; for although we can put a complete stop to many diseases, and medicine and diet are of great advantage in others, over which we have not so complete a dominion, no one can be blind to their inefficiency in many cases, and to the want of agreement among practitioners in physic, both in regard to the indications of cure and choice of means for fulfilling such indications.

"The system of arrangement which is here offered to the student, is founded on every circumstance attending diseases, which furnishes any hint as to their similitudes or dissimilitudes, and is therefore not merely historical, nor ætiological, nor anatomical, but is of a mixed nature, borrowing assistance from all these methods. It follows, at a humble distance, the celebrated works of Sauvages, and Dr. Cullen; for although Sauvages calls his nosological gical arrangement, an historical one, that is, one which is founded on the history and symptoms of disease alone, yet it is evident to any one who studies it, that he has taken into account, many other circumstances of analogy, and it would therefore appear vain and self-sufficient to pretend that the method here followed is new in its kind.

"But although I have adopted the principles which seem to have regulated both Sauvages and Cullen, it will be found that I have not strictly followed them in the execution of the subjsct; for it has appeared to me, as well as to many others, that Sauvages has not only subdivided too much, but has admitted many diseases as genera, and still more as species, some of which have no pretension to be considered as distinct diseases, and others of which are admitted in more places than one under different names.

"From the arrangement of Dr. Cullen, this table differs on another principle. His fondness for simplifying appears to have led him to generalize more than the nature of the subject justifies, and accordingly he has made too few general divisions or classes; for it will be found that some of his classes comprehend orders, and many orders comprehend genera, which have scarcely the most distant analogy. The just reputation and great learning however both of Sauvages and Dr. Cullen, and above all, the difficulty of the subject, ought to make every one extremely cautious of criticizing, far more of censuring their works.

"In a mere table such as the present one, it will not be expected that I should enter into a detail of those principles which regulate the division of diseases into classes, orders, genera, and species, and explain by what rules genera, species, and varieties are to be distinguished from each other. But when I reflect on the high degree of estimation in which the nosological arrangement of the celebrated Dr. Cullen is so justly held, and how much I have presumed to differ from so great and respectable an authority, I ought to feel it a duty incumbent on me to assign the reasons of my dissenting from him. In a few remarkable instances I have done so in notes, but the size and form of the table has prevented me from accomplishing this in every instance. Should the table fall into the hands of any medical men who are not students, the author intreats them to consider it as a mere guide in his lectures."

Our readers will be able to form an idea of this table from the following abstract of the Classes and Orders, viz.

CLASS I. PYREXIÆ, or Febrile diseases.

*Ord.* I. PHLEGMASIÆ, Inflammations.

*Gen.* I. PHLEG. sthenicæ.

Spec. 1. Cephalitis, &c. &c. 23 species.

*Gen.* 2. PHLEG. asthenicæ.

Spec. 1. Erysipelas, &c. 4 species.

*Ord.* II. FEBRES, Fevers.

Contains 11 genera and 38 species.

CLASS

CLASS II. HÆMORRHAGIÆ.

*Ord.* I. HÆM. ARTERIOSÆ, contains 6 genera and 12 species.

*Ord.* II. HÆM. VENOSÆ, same genera and species.

CLASS III. FLUXUS, Morbid Evacuations.
*Ord.* I. FL. cum febre, 4 genera and 8 species.
*Ord.* II. FL. sine febre, 3 genera and 6 species.

CLASS IV. NEUROSES, Nervous Diseases.
*Ord.* I. MORB. CONVULSIVI, 7 genera and 13 species.
*Ord.* II. SPASMI, 5 genera and 10 species.
*Ord.* III. COMATA, 5 genera and 10 species.
*Ord.* IV. ADYNAMIÆ, 7 genera and 15 species.
*Ord.* V. DOLORES, 11 genera and 21 species.
*Ord.* VI. VESANIÆ, 3 genera and 8 species.
*Ord.* VII. ERETHISMUS or morbid sensibility, 2 genera and 7 species.

CLASS V. INTUMESCENTIÆ, Swellings.

*Ord.* I. HYDROPES, dropsies, 6 genera and 13 species.

*Ord.* II. INTUM. ADIPOSÆ, 1 genus and 2 species.

*Ord.* III. INTUM. FLATUOSÆ, 2 genera and 4 species.

CLASS VI. CACHEXIÆ, Morbid Habit of Body.

*Ord.* I. CACH. ANTONICÆ; this order includes scrophula and morbid enlargements of the viscera.

*Ord.* II. CACH. CONTAGIOSÆ, lues, yaws, &c.

*Ord.* III. VITIA CACHECTICA, cutaneous diseases and mortifications.

CLASS VII. EPISCHESES, Retention of natural discharges, 9 genera and 21 species.

CLASS VIII. LOCALES.

*Ord.* I. DYSCINESIÆ INFLAMMATORIÆ.

*Ord.* II. DYSC. ATONICÆ.

*Ord.* III. HERNIÆ.

*Ord.* IV. PROLAPSUS.

*Ord.* V. LUXATIO.

*Ord.* VI. TUMORES.

*Ord.* VII. VULNUS.

*Ord.* VIII. ULCUS.

*Ord.* IX. FRACTURA.

The modesty of the author's preface disarms criticism, otherwise we would ask, why an order of *painful diseases* should have been admitted at all; and if admitted, why gout, stone, colic, cramp, &c. should have been excluded from it? These, and several other questions which may be asked, are, doubtless, answered in the lectures; and on the whole, we consider this arrangement as very well adapted to the purposes of teaching, and the practice of medicine.

MEDICAL

